# Australia for the Sons of Medical Men

**Published:** 1909-05-29

**Authors:** Richard Arthur

**Affiliations:** President Immigration League of Australasia


*To the Editor of* THE HOSPITAL.

### AUSTRALIA FOR THE SONS OF MEDICAL MEN.

*To the Editor of* The Hospital.

Sir,—Some time ago you were so good as to allow me to call attention in your columns to the opportunities which life on the land in Australia offered to lads and young men, and to point out what excellent avenues to this the various Government Agricultural Colleges were. At these institutions an admirable training in scientific and practical agriculture in all its branches can be obtained at a cost of from £20 to £30 a year, including first-class board and lodging. After finishing the course, fertile land in a district of good rainfall can be obtained on reasonable terms for either sheep, wheat, dairying, fruit growing, or mixed farming. As a result of this many doctors wrote to me, and some have already sent out their sons. I am at present in London and will be glad to correspond with any others, or arrange an interview with them. I have visited most of these colleges myself, and so can speak from experience. I hope to make arrangements for a lantern lecture on the Australian agricultural colleges, to be given at Belfast when the British Medical Association is meeting there, and to be present myself, to see any medical men who may desire further information. My address for the next two months will be the Royal Colonial Institute, Northumberland Avenue, W.C.

I am, yours truly,

Richard Arthur, M.D.,
President Immigration League of Australasia.

